# Appraisal of hydatidiform mole incidence and registration rates in Ireland following the establishment of a National Gestational Trophoblastic Disease Registry

**DOI:** 10.1136/jcp-2023-209270

**Published:** 2024-03-30

**Authors:** Caroline M Joyce, Craig Wakefield, Daphne Chen-Maxwell, Susan Dineen, Caitriona Kenneally, Paul Downey, Catherine Duffy, Keelin O'Donoghue, John Coulter, Brendan Fitzgerald

**Affiliations:** 1 Pregnancy Loss Research Group, Department of Obstetrics and Gynaecology, University College Cork, Cork, Ireland; 2 Department of Biochemistry and Cell Biology, University College Cork, Cork, Ireland; 3 INFANT Research Centre, University College Cork, Cork, Ireland; 4 Department of Pathology, Cork University Hospital, Cork, Ireland; 5 Department of Obstetrics and Gynaecology, Cork University Maternity Hospital, Cork, Ireland; 6 Department of Pathology, National Maternity Hospital, Dublin, Ireland; 7 HSE National Cancer Control Programme, Dublin, Ireland

**Keywords:** Medical Oncology, Pathology, Molecular, PREGNANCY, DIAGNOSIS, GENETICS

## Abstract

**Aims:**

This study aimed to re-evaluate the incidence of hydatidiform mole (HM) and determine gestational trophoblastic disease (GTD) registration rates in Ireland following the establishment of the National GTD Registry in 2017.

**Methods:**

We performed a 3-year retrospective audit of HM cases (January 2017 to December 2019) reported in our centre. In 2019, we surveyed Irish pathology laboratories to determine the number of HMs diagnosed nationally and compared this data to that recorded in the National GTD Registry. Additionally, we compared both local and national HM incidence rates to those reported internationally.

**Results:**

In the 3-year local audit, we identified 87 HMs among 1856 products of conception (POCs) providing a local HM incidence rate of 3.92 per 1000 births. The 1-year pathology survey recorded 170 HMs in 6008 POCs, yielding a national incidence rate of 2.86 per 1000 births. Importantly, the local HM incidence rate exceeded the national incidence rate by 37% and the local partial HM incidence (1 in 296 births) was 64% higher than the nationally incidence rate (1 in 484 births). Notably, 42% of the HM and atypical POCs diagnosed nationally were not reported to the National GTD Registry.

**Conclusions:**

Our study reveals increased HM incidence rates both locally and nationally compared with previous Irish studies. The higher local PHM incidence may reflect more limited access to ploidy analysis in other pathology laboratories nationally. Significantly, almost half of the women with diagnosed or suspected HM were not registered with the National GTD Centre.

**WHAT IS ALREADY KNOWN ON THIS TOPIC**

- Access to accurate hydatidiform mole (HM) incidence rates is hindered by lack of standardised data collection, the absence of a universal denominator and limited national registration centres worldwide which makes comparative analysis of disease prevalence and treatment outcomes more challenging.

**WHAT THIS STUDY ADDS**

- This study demonstrates how advances in pathological techniques have improved HM diagnosis, resulting in higher incidence rates in centres with access to ancillary diagnostic techniques. It also highlights the issue of low registration rates with our National Gestational Trophoblastic Disease Centre, which compromises the reliability of registry data.

**HOW THIS STUDY MIGHT AFFECT RESEARCH, PRACTICE OR POLICY**

- This study emphasises the need to re-evaluate HM incidence rates given recent advances in pathological diagnosis. It highlights the importance of adopting a universally accepted denominator for accurate HM incidence rates and advocates for mandatory registration to ensure equitable access for all women to expert clinical management of trophoblastic disease.

## Introduction

 Gestational trophoblastic disease (GTD) encompasses a range of conditions occurring during or after pregnancy. It is classified by the World Health Organisation (WHO) into two pre-malignant (complete hydatidiform mole, CHM and partial hydatidiform mole, PHM) and four malignant disorders (invasive hydatidiform mole, choriocarcinoma, placental site trophoblastic tumour, PSTT and epithelioid trophoblastic tumour, ETT).^1^ A recently proposed trophoblastic lesion, atypical placental site nodule, is considered a precursor to ETT.^2 3^ Accurate subclassification hinges on the identification of distinct pathological and genetic profiles, often necessitating histopathological examination complemented by ancillary techniques.^4^ Early detection and accurate diagnosis of GTD coupled with effective, low toxicity chemotherapy affords cure rates approaching 100%.^5^

Hydatidiform Mole (HM) represents the most common GTD manifestation and is classified into two genetically distinct entities: CHM characterised by two haploid sets of paternally derived genes (androgenetic diploid) and PHM characterised by one excess haploid set of paternal genes (diandric triploid).^6^,^8^ Ultrasonography in early pregnancy facilitates HM detection, often before 10 weeks gestation when classical histological features are less apparent.^9^ In rare instances, a woman may experience more than one CHM due to familial recurrent CHM which is associated with a diploid biparental molar conceptus.^10^ Following molar pregnancy, the risk of developing persistent GTD is estimated at 15%–20% for CHM and 0.5%–5% for PHM.^11^ While most cases of persistent GTD occur within 12 weeks of diagnosis, exceptionally longer latency periods have been documented.^12^

Historically, GTD epidemiological data has been limited by inconsistencies in the application of diagnostic criteria, lack of access to specialist diagnostic services, the increasing availability of medical termination and non-standardised reporting of incidence rates.^13^ Established risk factors include extreme maternal age and prior molar pregnancies, with additional risk factors potentially linked to diet and low socioeconomic status.^14^ A distinct geographical distribution is observed, with the highest incidence in Southeast Asia (10 per 1000 pregnancies) and lower rates in Western Europe and North America (1–2 per 1000 pregnancies).^15 16^

Accurate subclassification of molar and non-molar specimens is essential for risk stratification and clinical management.^17 18^ Even within specialist referral centres with experienced pathologists using consensus morphological diagnostic criteria, up to 20% of HMs are misclassified.^19^ These diagnostic challenges likely influence the HM incidence rates reported. Use of ancillary techniques such as p57 immunohistochemistry (IHC), ploidy analysis (in situ hybridisation, ISH and flow cytometry) and molecular genotyping, in conjunction with morphological assessment, has improved HM diagnostic accuracy.^20^

Use of p57 IHC, an inexpensive and reproducible adjunct diagnostic test, has greatly improved the histological discrimination of CHM from its mimics.^21^ In androgenetic CHMs staining for p57 is lost in villous cytotrophoblast and stromal cells. Rarely a mixed pattern of positive and negative staining is observed in villous cytotrophoblasts and stromal cells in a phenomenon termed p57 discordant villi, typically associated with androgenetic/biparental mosaic conceptions.^22^ In addition, DNA ploidy analysis using ISH or flow cytometry may be integrated with morphological assessment to differentiate diploid (CHM or non-molar) from triploid (PHM) conceptions. Ploidy analysis will not differentiate between diandric PHM triploidy and digynic non-molar triploidy; however, this may not be a practical issue that leads to overdiagnosis of PHM.^23 24^ Molecular genotyping using short tandem repeats (STRs) will identify the parental genetic contribution and can infer ploidy allowing accurate classification of molar and non-molar pregnancies. Nevertheless, the cost and scientific experience required for genomic analysis has largely limited its widespread adoption in pathology laboratories.

While GTD is the most curable of all gynaecologic malignancies, its comparative rarity has led to inconsistencies in clinical management.^25^ For low risk, non-metastatic cases, nearly all patients can expect to be cured and many with advanced disease can achieve remission with appropriate regimens without negatively affecting their reproductive status.^26^ Studies have shown that once serum human chorionic gonadotrophin (hCG) levels normalise, the risk of developing gestational trophoblastic neoplasia (GTN) is very low.^27^,^29^ International experience, most notably that of the UK, has shown that outcomes can be significantly improved with centralised disease registration and hCG monitoring.^30^ This allows early specialist input, early detection of disease progression and enables use of effective, low toxicity chemotherapy regimens.^31^,^33^

In Ireland, the National Cancer Control Programme (NCCP) established a National GTD Registry, Monitoring and Advisory Centre in 2017 to provide multidisciplinary care for this rare disease.^34 35^ Women with confirmed or suspected GTD are voluntarily registered with the centre for monitoring and follow-up care based on national clinical guidelines.^25 30^

This study aims to establish local rates of HM diagnosis, determine the number of GTD cases reported nationally, evaluate access to ancillary diagnostic techniques in Irish pathology laboratories, determine registration rates with the National GTD Centre and derive Irish national HM incidence rates.

## Materials and methods

### Study design

Two pathology studies were carried out to assess the incidence of HM in Ireland. The first was a local pathology audit at Cork University Hospital (CUH) from 2017 to 2019, and the second was a national pathology survey involving all 33 pathology laboratories in Ireland, focusing on cases diagnosed in 2019.

### Local pathology audit

We conducted a retrospective review of GTD cases reported in the pathology laboratory at CUH from January 2017 to December 2019. Study data was collected by interrogating the laboratory database to identify products of conception (POCs) examined and HM cases reported during the study period. The pathology laboratory at CUH is co-located with a large tertiary maternity hospital (Cork University Maternity Hospital, CUMH) handling approximately 8000 births annually. The laboratory has access to p57 IHC and an adapted *HER2* dual-colour dual-hapten ISH (DDISH) assay for ploidy analysis, to aid in HM diagnosis.^36^ The pathology laboratory at CUH is accredited to ISO 15189 international standards by the Irish National Accreditation Board. During the study period, *HER2* DDISH staining quality was monitored by internal quality assessment and by participation in two external quality assurance schemes (UK NEQAS and NordiQC) and assay performance was deemed satisfactory in both.

### National pathology survey

The national pathology survey was conducted using a questionnaire designed by a multidisciplinary team comprising members of the steering committee of the National GTD Centre. The questionnaire contained 21 questions which sought to determine the number of POCs examined and GTD cases reported in 2019 (online supplemental material). Distribution of the survey to laboratory managers in the 33 Irish pathology laboratories was co-ordinated by the NCCP. The survey sought information on POC case volume and rates of GTD diagnoses. Additionally, it sought information on indeterminate cases, where a pathology report indicated that an ‘HM could not be excluded’ or where cases were ‘suspicious for HM’ without a conclusive diagnosis of partial or complete mole. These cases are recorded as ‘atypical POCs’ in data collection spreadsheets. The survey also collected data on the number of cases with p57 discordant villi.

Respondents were asked to provide details of access to ancillary techniques (p57 immunostaining, DNA ploidy and STR genotyping) within their laboratories. Laboratories were also questioned about their adherence to guidance from the Faculty of Pathology of the Royal College of Physicians of Ireland (RCPI) on reporting GTD cases. The Faculty of Pathology recommends inclusion of the following comment on all GTD reports, “Patient registration with the National Gestational Trophoblastic Centre is recommended”. This latter comment aims to promote patient registration in accordance with the National GTD clinical guidelines.^25^

### GTD registration and HM incidence rates

Data provided in the questionnaire responses from Irish pathology laboratories was cross-referenced with cases reported to the National GTD Registry in 2019. This allowed us determine registration rates and identify potential gaps in the registration process. The data collected from our local pathology audit and the national pathology survey was compared with previously published Irish and International incidence rates to identify worldwide trends in the prevalence of GTD.

### Statistical analysis

Incidence rates in the local audit and national pathology survey were calculated per 1000 births. We used ‘total births’ as our denominator and define this as the number of live births and stillbirths per annum.^37^ We referenced the South/Southwest Hospital Group Annual Reports to establish the total births in CUMH from 2017 to 2019.^38^,^40^ The national Irish birth rate was taken from the Healthcare Pricing House report for 2019.^37^ All data analyses were performed using Microsoft Excel 2021.

## Results

### Local pathology audit

**Figure 1:**
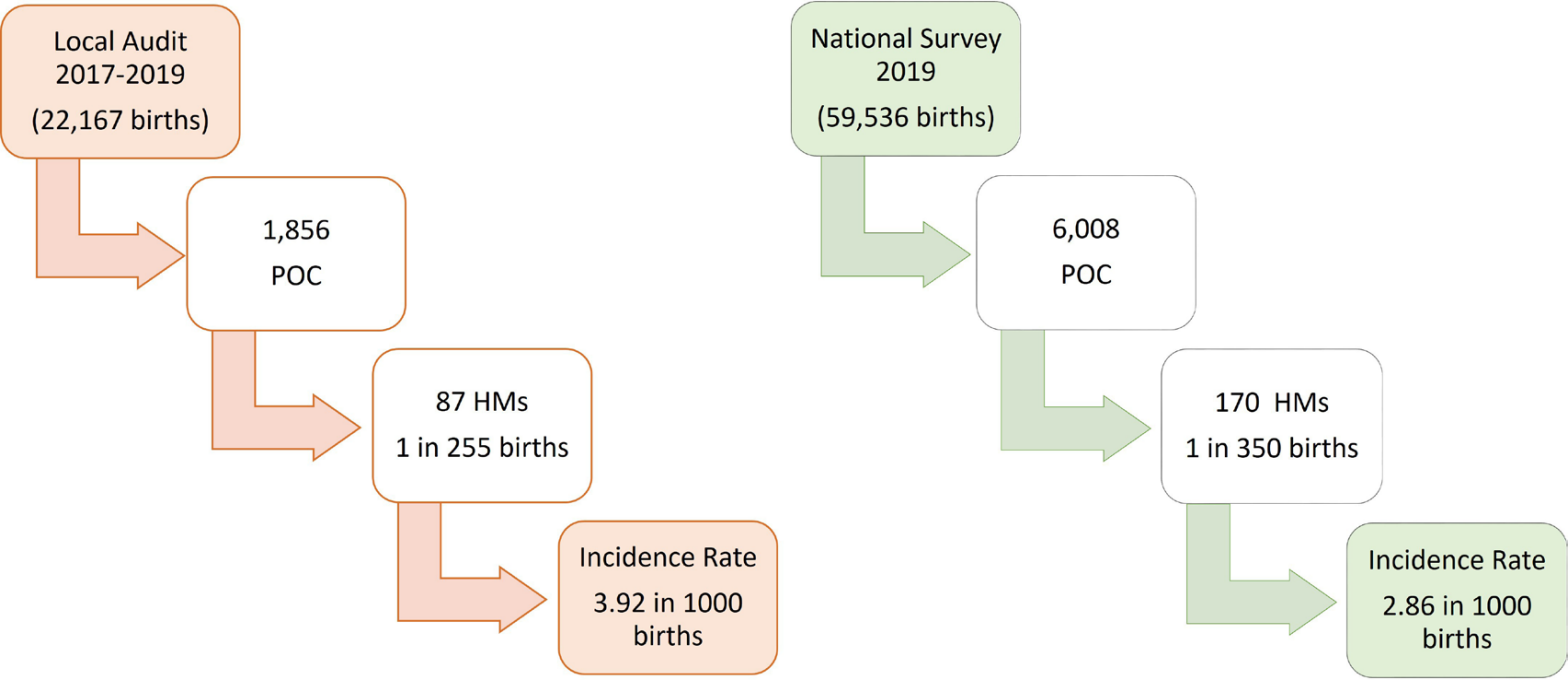
Local and national hydatidiform mole (HM) incidence rates. National birth rate data was sourced from the Healthcare Pricing Office. Local birth rate data was sourced from the South/Southwest Hospital Group’s annual reports 2017–2019. Births=total births (live birth and stillbirth). POC, product of conception.

**Figure 2:**
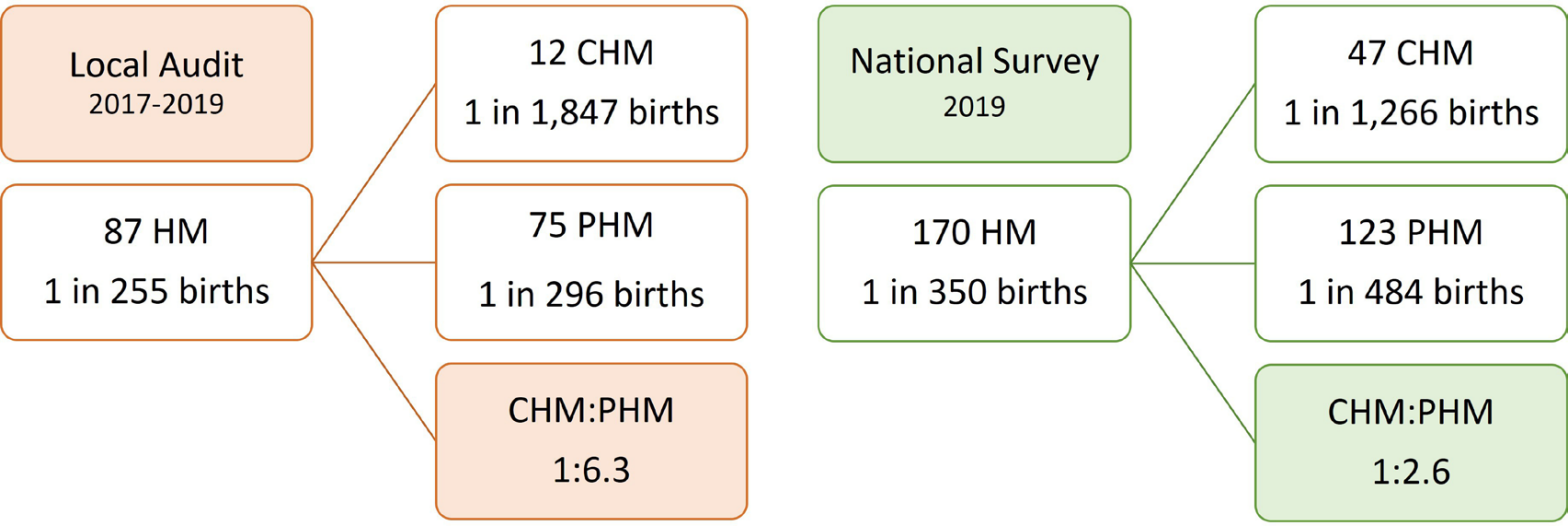
Ratio of complete to partial hydatidiform mole (HM) in the local audit and national survey. CHM, complete HM; PHM, partial HM.

The local CUH 3-year pathology audit identified 87 HMs in 1856 POCs (figure 1). This provided an incidence rate of 3.92/1000 births. In total, we identified 12 CHMs and 75 PHMs equating to 1/1847 and 1/296 births, respectively. This gives a CHM:PHM ratio of 1:6.3 (figure 2). In the local Cork audit (2017–2019), *HER2* DDISH was requested on 151 POCs which confirmed triploidy in 66 (43.7%) and diploidy in 81 (53.6%) cases. In total, 4 cases were equivocal (2.6%) and 9 PHMs were reported without *HER2* DDISH ploidy analysis, giving a total of 75 PHMs.

### National pathology survey

The 2019 national pathology survey identified 170 HMs in 6008 POCs (figure 1). This provided an HM incidence rate of 2.86/1000 births. There were 47 CHMs and 123 PHMs identified during this period giving incidence rates of 1/1266 and 1/484 births, respectively. This yielded a CHM to PHM ratio of 1:2.6 (figure 2). Most laboratories (58%, 11/19) analysing POCs had access to p57 immunostaining, two laboratories had access to ploidy analysis (Silver ISH,SISH and DDISH) and there was no laboratory with access to molecular genotyping (table 1). Five hospital laboratories handled 62% (n=3705) of the workload and four of these hospitals had perinatal pathologists examining and reporting POCs. From the survey responses and follow-up phone calls, we determined that all histopathology laboratories who process POCs responded to the survey giving a 100% response rate (19/19).

**Table 1:** Results from the National Pathology Survey 2019

	Classification/n (%)	
GTD diagnosis (total POC=6008)		
HM and atypical POCs	197 (3.3%)	
CHM	47 (0.8%)	
PHM	123 (2.0%)	
Atypical POCs (suspicious of HM)	27 (0.4%)	
APSN	1 (0.02%)	
p57 discordant villi	1 (0.02%)	
Gestational choriocarcinoma	3 (0.05%)	
PSTT/ETT	0 (0%)	
Access to ancillary techniques*	Yes/n (%)	No/n (%)
p57 IHC	11 (58%)	8 (42%)
Ploidy analysis	2 (11%)	17 (89%)
STR genotyping	0 (0%)	19 (100%)
Faculty of Pathology recommendation on reports†	3 (16%)	16 (84%)

* Reponses from the 19 pathology laboratories who processed products of conception (POCs).

† Faculty of Pathology recommended comment on GTD reports, “Patient registration with the National GTD Centre is recommended”.

APSN, atypical placental site nodule; CMH, complete HM; ETT, epithelioid trophoblastic tumour; GTD, gestational trophoblastic disease; HM, hydatidiform mole; IHC, immunohistochemistry; n, number; PHM, partial HM; PSTT, placental site trophoblastic tumour; STR, short tandem repeat.

### GTD registration and HM incidence rates

**Figure 3:**
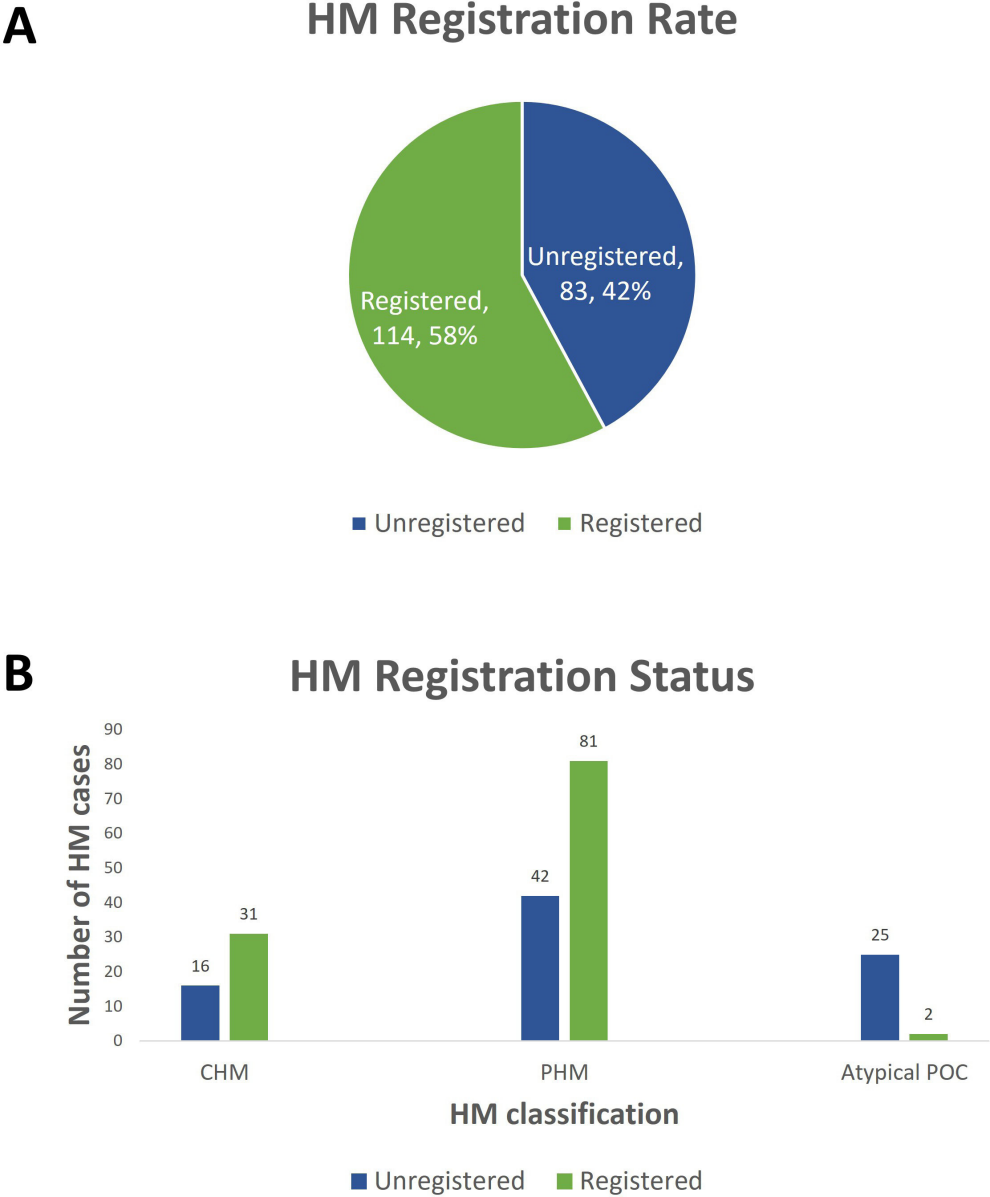
Hydatidiform mole (HM) registration rates with the National GTD Registry in 2019. (A) Pie chart showing the total number of HM cases registered and (B) bar chart showing the subcategories of HM registered and unregistered. CHM, complete HM; PHM, partial HM; POC, product of conception.

A comparison of GTD cases reported in the national pathology survey to those registered with the National GTD Registry in 2019 revealed that 42% of diagnosed/suspected HM cases (83/197) were unregistered. All choriocarcinoma cases reported were registered but 34% of women with CHM (16/47) were unregistered. In addition, 34% of women with PHM (42/123) and 93% of women with ‘atypical’ POCs (25/27) were unregistered (figure 3). A review of the GTD cases registered in 2019 found that 6.5% (2/31) of the CHMs and none of the PHMs required follow-up chemotherapy.

The local HM incidence rate is 33% higher (3.92/1000) than the rate reported in the national pathology survey (2.86/1000). While the CHM incidence is consistent across both studies, the local PHM incidence (1 in 296) surpasses the national rate (1 in 484). For a comprehensive comparison of incidence rates from this study with previous Irish studies, please refer to table 2. Additionally, a comparison with international studies is detailed in table 3.

**Table 2:** Comparison of all Irish HM incidence rates

Parameter	Dublin 3-year audit* (1988–1990)	Cork 3-year audit (2017–2019)	Dublin 10-year audit† (1997–2006)	National Pathology Survey 2019
Denominator	19 457 Pregnancies	22 167 Total births	66 296 Total births	59 536 Total births
POC	2251	1856	N/A	6008
HM	38	87	262	170
HM incidence	1.95 in 1000 1 in 521	3.92 in 1000 1 in 255	3.95 in 1000 1 in 253	2.86 in 1000 1 in 350
CHM	10	12	60	47
CHM incidence	1 in 1945	1 in 1846	1 in 1105	1 in 1266
PHM	28	75	202	123
PHM incidence	1 in 695	1 in 296	1 in 328	1 in 484
CHM:PHM ratio	1:2.8	1:6.3	1:3.4	1:2.6

* Jeffers *et al*.^41^

† Purandare *et al.*^42^

CHM, complete HM; HM, hydatidiform mole; N/A, not available; PHM, partial HM; POC, product of conception.

**Table 3:** Recent studies on HM incidence rates

Author	Country	Period/years	Incidence per 1000	Denominator	CHM:PHM ratio
Present study	Ireland	2019	2.86	Total Births	1:2.6
Present study	Cork	2017–2019	3.92	Total Births	1:6.3
Eysbouts *et al*^13^	The Netherlands	1994–2013	1.67	Deliveries	1:1.3*
Colgan *et al*^50^	Canada	2013–2015	3.3	Deliveries	1:1.7
Lund *et al*^57^	Denmark	2007–2014	1.79 1.23	Deliveries Pregnancies	1:1
Tham *et al*^15^	North England, North Wales	1991–1999	1.33	Live births	1:1.6
Savage *et al*^11^	England and Wales	2000–2009	1.65	Viable conceptions	1:1.3*
Joneborg *et al*^58^	Stockholm County, Sweden	1991–2010	2.08 1.48	Deliveries Viable conceptions	1:0.8*
Matsui *et al*^59^	Japan	1985–2002	1.5	Viable conceptions	1:0.8
Yuk *et al*^60^	South Korea	2009–2015	1.10	Pregnancies	Not available

Adapted from Lund *et al.*^57^

* CHM:PHM ratio calculated from the study data provided in the research study.

CHM, complete hydatidiform mole; PHM, partial hydatidiform mole.

## Discussion

This is the first study to gather data on national HM incidence rates and GTD reporting practices following the establishment of the Irish National GTD Registry in 2017.

Our local 3-year pathology audit in Cork revealed a HM incidence rate of 1 in 255 births (3.92 in 1000) which is nearly double the rate reported in a similar 3-year Irish study in Dublin (1 in 512 pregnancies).^41^ While both studies reported similar CHM rates, our study found a significantly higher PHM incidence rate at 1 in 296 births, almost 60% higher than the Dublin study. Both studies employed ploidy techniques to assist PHM diagnosis (flow cytometry in Dublin and *HER2* DDISH analysis in Cork). However, the Dublin study used ‘pregnancy’ as denominator without defining ‘pregnancy’, which limits an accurate comparison between both studies.

Another 10-year Dublin-based study reported an HM incidence rate of 1 in 253 live births, which closely resembles our local rate (1 in 255 births) although denominators differed. In terms of CHM incidence rates, the Dublin study reported higher rates (1 in 1105 live births) compared with our local audit, but PHM incidence rates were similar (1 in 328 live births). Ancillary techniques (p57 IHC and flow cytometry in Dublin) were used to aid morphological diagnosis in both studies.^42^ Our local audit reported a CHM:PHM ratio of 1:6.3, indicating a higher prevalence of PHM (relative to CHM). This is consistent with higher PHM rates reported in similar Irish and UK studies although at a lower proportion (table 3).^1541^,^45^

The National Pathology Survey yielded an HM incidence rate of 1 in 350 births (2.85 in 1000), at least a third lower than our local incidence (1 in 255 births). In addition, the local incidence showed a higher prevalence of PHM (CHM:PHM ratio of 1:6.3) compared with the national survey (CHM:PHM ratio of 1:2.6). This discrepancy may reflect local reporting practice whereby experienced perinatal pathologists have access to *HER2* DDISH ploidy to aid PHM diagnosis.^36^ The liberal use of ploidy analysis locally, where it was applied in the examination of 151 of 1856 (8.1%) of POCs and the lack of access to ploidy in all but one centre outside of Cork, may contribute to the identification of a higher incidence of PHM in Cork. In the Cork audit (2017–2019) when the *HER2* DDISH assay was used to assist with PHM diagnosis, not too dissimilar numbers of diploid (53.6%, 81/151) and triploid (43.7%, 66/151) conceptuses were identified. This finding reflects and emphasises the difficulty that exists when trying to diagnose PHM with morphology alone. Information on the pattern of use of ploidy analysis in the only other centre with access to it nationally was not available. Questions raised by variations in diagnostic rates in the survey suggest that data should be collected in future national surveys not just on technique availability but on patterns of use (such as numbers of tests performed as a percentage of POCs received or as an expression relative to the number of HM diagnosed). In total, 27 cases of ‘atypical POC’ were recorded in the national survey in comparison to 170 conclusive HM diagnoses. The National GTD Centre can use this information, together with survey data on variation in reported incidence rates nationally, to inform discussions on how diagnostics can be improved and increasingly standardised nationally.

Variations in diagnostic rates may reflect differences in the application of morphologic criteria, access to adjunct diagnostic techniques and the considerable morphological overlap in early gestation between CHM, PHM and non-molar pregnancies (especially those with aneuploidy).^46 47^ Use of ancillary techniques to aid morphological examination has vastly improved the diagnosis of PHM and resulted in an increased incidence of PHM relative to CHM in recent years.^13^ This highlights the importance of incorporating ploidy analysis into routine practice when evaluating POCs.

The low adherence to national clinical GTD guidelines and RCPI Faculty of Pathology guidance on patient registration with the National GTD Centre and use of p57 IHC for POCs highlights the need for improved standardisation of reporting practice.^25 30^ Results from the national survey show that in laboratories processing greater than 10 POCs annually, 29% (4/14) did not have access to p57 IHC and 86% (12/14) did not recommend patient registration with the National GTD Centre.

Our gap analysis of the GTD registration process in Ireland revealed suboptimal registration rates with the National GTD Registry, with nearly half (42%, 83/197) of eligible women remaining unregistered. This lapse in registration likely poses clinical risks, especially since 34% (16/47) of women with CHM were unregistered, a condition associated with a higher risk of persistent GTD, necessitating close clinical surveillance. Effective management of GTD relies on a multidisciplinary approach and the registration of patients with expert centres, which is increasingly seen to represent a minimum standard of care.^25 30^ Notably, the UK has a nationally coordinated GTD programme in place since the 1970s, contributing to impressive long-term survival outcomes.^30^ Centralised registration offers opportunities to establish national incidence rates, conduct clinical audits and assess service quality. While voluntary registration may improve with heightened awareness and commitment from healthcare professionals, some expert centres advocate for mandatory registration due to challenges associated with voluntary compliance.^34^

The disparities in HM incidence rates worldwide are likely attributable to inconsistencies in data collection methods, the utilisation of different denominators for reporting and variations in access to adjunct diagnostic techniques. Our study’s findings of increased HM incidence rates are generally consistent with those reported in other European, Scandinavian and North America countries.^13 48 49^ For instance, in the Netherlands, a population-based study spanning two decades revealed an increase in incidence rates during the first 10 years, particularly for PHM. Subsequently, the incidence rates stabilised, resulting in an overall HM incidence of 1.67 per 1000 deliveries. This trend was accompanied by a decrease in the number of atypical POCs reflecting advancements in diagnostic techniques.^13^ In Canada, a hospital-based retrospective review of HM incidence over a 27-month period incorporating molecular genotyping to aid diagnosis reported an incidence of 3.3 per 1000 deliveries. This figure corroborates our HM incidence rates, locally (3.92 per 1000 live births) and nationally (2.86 per 1000 live births).^50^ Notably, the CHM:PHM ratio in the Canadian study (1:1.7) shows a lower PHM prevalence than ours but the Canadian study is a much smaller study with 49 HMs (18 CHM and 31 PHM).

Our study has shown that our local PHM incidence rate exceeds both national and international figures. While one might argue that this disparity could be attributed to an overdiagnosis of PHM in our local setting, it should be noted that our adapted *HER2* DDISH ploidy assay did not overdiagnose PHM when audited by molecular genotyping.^23^ In terms of a population bias accounting for differences in incidence rates, we did not observe births to younger mothers in Ireland where the average maternal age was 32.5 years compared with the European average (30.6 years). In terms of ethnicity, the majority of babies (77%) were born to mothers of Irish nationality. The use of assisted reproductive techniques (ART) is unlikely to have affected HM incidence rates as ART only received public funding in Ireland in 2022 and its availability during the study period was limited to private fertility service providers. Of note, there is no national register of miscarriages in Ireland, so it is difficult to capture all fetal losses.^51^ However, the national perinatal mortality report does not identify an increase in the stillbirth rate in our local population (4.0 per 1000 births) compared with the national stillbirth rate (4.06 per 1000 births) when corrected for congenital anomalies.^40 52^

Realistically, one would anticipate a higher prevalence of PHM compared with CHM, given that a PHM can occur regularly throughout a woman’s reproductive life, whereas CHM is typically age related.^11^ Historically, CHM:PHM ratios of 1:3 have been reported in the UK.^45 53^ Given our results, we suspect PHMs are underdiagnosed nationally and internationally. This raises the possibility that some instances of GTD, which follow seemingly ‘normal pregnancies’, may be linked to undiagnosed PHM. This lends support to the notion that persistent GTD is a rare occurrence following a first-trimester pregnancy loss unrelated to molar pregnancy.^50 54^ Furthermore, a recent systematic review found that the progression of PHM to GTN is an exceptionally rare event, occurring in less than 1% of cases.^55^ Consequently, the potential underdiagnosis of PHM is unlikely to have a substantial impact on clinical outcomes given the low risk of malignant progression.

Our study has certain limitations, including our reliance on the proficiency of laboratories to extract cases from their databases and accurately interpret pathology coding. Additionally, patient characteristics such as ethnicity and maternal age, which could potentially influence incidence rates, were not collected. Moreover, pregnancy losses that did not undergo histopathological examination were not included in our study.

Accurate HM incidence rates are not readily available worldwide due to a lack of central registries and under-reporting of cases to established registries.^56^ The comparison of GTD incidence is further complicated by the use of different denominators.^16^ While the ‘total number of conceptions’ is the most appropriate denominator for reporting incidence, this is virtually impossible to establish with absolute certainty. Consequently, surrogate measures are employed, leading to variable reporting based on the total number of pregnancies (which, like conceptions, is difficult to define), deliveries, total births or live births. Additionally, there is variable access to ancillary techniques to aid HM differential diagnosis worldwide, with some centres relying solely on morphological criteria for diagnosis. This divergence in pathology reporting practices has resulted in inconsistent incidence rates. Given the advancements in obstetric management and pathological diagnosis over the last two decades, a re-evaluation of HM incidence rates is warranted worldwide.

## Conclusion

This study provides the first audit of national GTD data in Ireland. Our findings suggest a potential underdiagnosis of PHM nationally and internationally. They emphasise the need to adopt standardised diagnostic reporting of HMs using specific morphological criteria and ancillary techniques. Furthermore, this study advocates for mandatory registration to guarantee equitable access for all women to expert clinical care in a trophoblastic disease centre.

## Supplementary material

10.1136/jcp-2023-209270 online supplemental file 1

## Data Availability

All data relevant to the study are included in the article or uploaded as supplementary information.
