# Supplementary material for: Appraisal of hydatidiform mole incidence and registration rates in Ireland following the establishment of a National Gestational Trophoblastic Disease Registry
Source: J Clin Pathol. 2024 Mar 30;78(12):e209270. doi: 10.1136/jcp-2023-209270 (PMC12703346; doi:10.1136/jcp-2023-209270)
Supplement: online supplemental file 1 [file jcp-78-12-s001.pdf]

National Pathology Survey 2019

**For the year 2019 could you please complete the questions below with numbers from your laboratory?**		
**Laboratory Name:**		
How many Products of Conception did your laboratory examine in 2019?		
How many Complete Hydatidiform Moles did you diagnose in 2019?		
How many Partial Hydatidiform Moles did you diagnose in 2019?		
How many cases of 'Atypical' products of conception were reported? [These would include cases where a "hydatidiform mole could not be excluded" or where a case is "suspicious for hydatidiform mole" but a firm diagnosis of partial mole or complete mole could not be made].		
How many cases of "P57-discordant villi" did you diagnose in 2019?		
How many Gestational Choriocarcinomas did you diagnose in 2019?		
How many Atypical Placental Site Nodules did you diagnose in 2019?		
How many Placental Site Trophoblastic Tumours/ Epithelioid Trophoblastic Tumours did you diagnose in 2019?		
Do you have P57 immunohistochemistry within your laboratory?		Yes ( )<br>No ( )
	If yes, is the stain accredited?	Yes ( )<br>No ( )
	If you don't have P57, are you aware that the Faculty of Pathology recommends that you have access to P57 if you report products of conception?	Yes ( )<br>No ( )
	Do you plan to introduce P57 to your laboratory?	Yes ( )<br>No ( )
	If so, when?	
Do you have in house Ploidy Analysis for triploidy detection?		Yes ( )<br>No ( )
	If yes, what type?	
	If yes, is the test accredited?	
Do you have in house Molecular Genotyping for trophoblastic disease?		Yes ( )<br>No ( )
	If yes, is the test accredited?	Yes ( )<br>No ( )
If a second opinion or additional testing is needed what laboratory or laboratories do you refer your cases to?		
	How many external referrals did you send in 2019 for this purpose?	
When reporting molar pregnancies or cases suspicious for molar pregnancy do you add the following recommendation, that was endorsed by the Faculty of Pathology, to your laboratory report "Patient registration with the National Gestational Trophoblast Disease Centre is recommended?		Yes ( )<br>No ( )
General Comments		
